# E-MDD: an evidence-guided explainable framework for EEG-based depression recognition

**DOI:** 10.3389/fdgth.2026.1950514

**Published:** 2026-10-15

**Authors:** Junfen Huang, Zhuhuayang Zhang, Yingyu Cao, Zhenxi Zhao, Liangfei Niu

**Affiliations:** 1 College of Mechanical Engineering, Beijing Institute of Petrochemical Technology, Beijing, China; 2 College of Mechanical Engineering, Tiangong University, Tianjin, China

**Keywords:** deep learning, electroencephalography, evidence-guided explainability, large language models, major depressive disorder

## Abstract

**Introduction:**

An evidence-guided explainable framework, E-MDD, is developed to integrate high-dimensional EEG representations, neurophysiological biomarkers, and large language models for the recognition of EEG-based depression with concise and evidence-based textual explanations.

**Methods:**

The proposed E-MDD framework consists of three stages: classification, evidence construction, and evidence-guided inference. In the classification stage, two parallel feature extraction branches are employed to extract Electroencephalography latent features and neurophysiological biomarkers, which are subsequently fused and fed into a lightweight multilayer perceptron for the recognition of depression. In the evidence construction stage, the latent feature dimensions of the EEG are aligned with neurophysiological biomarkers, and the model's decision basis is identified to construct structured evidence supporting the classification results. In the evidence-guided inference stage, structured evidence and the proposed evidence-guided prompt are used to guide the large language model in generating concise textual explanations.

**Results:**

The experimental results on the Mumtaz dataset show that E-MDD achieves an AUC of 0.8472 and an F1-score of 0.8421. Meanwhile, the generated short-text explanations can effectively describe the basis of the model's decision-making for its predictions, demonstrating a high degree of faithfulness. The proposed framework also demonstrates consistent evidence-guided explanation performance across EEGNet and EEGConformer.

**Discussion:**

Overall, this study proposes a neurophysiologically interpretable framework for EEG-based deep learning models, which effectively enhances the interpretability of deep learning models for depression detection and provides a new perspective for understanding their decision-making mechanisms.

## 1 Introduction

Major Depressive Disorder (MDD) is one of the most prevalent and burdensome mental disorders worldwide, imposing profound impacts on functional capacity, increasing the risk of suicide, and placing a substantial burden on healthcare systems and society (1). The current diagnosis of depression relies heavily on subjective clinical interviews and symptom rating scales rather than objective biological biomarkers, leading to reported rates of missed diagnosis and misdiagnosis ranging from 30% to 50%.

Electroencephalography (EEG), due to its non-invasive nature, millisecond temporal resolution, and the ability to capture real-time neuronal electrical activity, has emerged as a promising objective modality for the identification of depression (2). Previous studies have shown that EEG signals in patients with depression exhibit abnormal neurophysiological patterns associated with depression (3–6). In recent years, machine learning and deep learning methods have been widely used to extract depression-related neurophysiological and spatiotemporal features from EEG signals, resulting in remarkable performance in automated depression recognition (7–10). However, most conventional machine learning and deep learning models operate as black-box systems, providing limited insight into their internal decision-making processes (11, 12). Consequently, learned representations and prediction results are difficult to interpret in terms of underlying neurophysiological mechanisms, preventing the establishment of meaningful links between model decisions and depression-related neural biomarkers (13). With the rapid advancement of large language models (LLMs), the integration of LLMs with EEG analysis has become an emerging frontier in intelligent brain disorder research, enabling the development of novel EEG-to-large language model(LLM) frameworks for representation learning, reasoning, and interpretable analysis (14, 15). Building upon pre-trained architectures such as BERT and GPT, recent studies have developed general-purpose EEG foundation models, including EEGPT and LaBraM (16, 17). Meanwhile, lightweight optimization techniques, including model pruning and few-shot prompting, have facilitated accurate emotion recognition and automated clinical documentation. EEGAgent employs a LLM as the central orchestrator, enabling end-to-end automated EEG analysis and diagnostic report generation.Furthermore, multimodal LLM frameworks that integrate heterogeneous physiological signals provide a promising approach for the auxiliary screening of depression and other psychiatric disorders (18–20). Despite recent progress, LLM-based EEG analysis still faces a critical challenge in interpretability. Most existing methods rely on black-box reasoning and do not establish explicit links between EEG spatiotemporal representations, depression-related neurophysiological mechanisms, and clinically meaningful descriptions. As a result, a dedicated semantic mapping framework for interpretable depression analysis remains absent (20).

To address these limitations, we propose an interpretable EEG analysis framework for depression that deeply integrates latent EEG representations, quantitative neurophysiological biomarkers, and the natural language reasoning capability of large language models. The proposed framework extends the conventional data-driven classification paradigm by mapping the decision-relevant dimensions in the latent space of the model to quantifiable neurophysiological biomarkers and subsequently translating these biomarkers into evidence-guided short-text explanations using a LLM. This provides a physiologically grounded approach to improving the interpretability of EEG-based deep learning models for the detection of depression.

The main contributions of this study are summarized as follows:

1. A Shapley Additive Explanations based feature attribution strategy is proposed to quantitatively assess the contribution of individual dimensions in high-dimensional EEG representations to the prediction of depression. The proposed strategy effectively mitigates feature redundancy and resolves the difficulty of quantifying feature importance in high-dimensional EEG representations, enabling fine-grained traceability and interpretation of model predictions at the feature level.
2. A representation alignment mechanism is proposed to establish precise semantic associations between high-dimensional EEG representations and neurophysiological biomarkers. By explicitly linking individual latent dimensions to depression-related neurophysiological indicators, the proposed mechanism addresses the limited interpretability of deep learning representations and bridges the gap between learned features and their underlying neurophysiological mechanisms, thereby allowing the transformation of latent model representations into explicit neurophysiological semantics.
3. An evidence-guided inference framework is developed for interpretable EEG-based depression analysis, together with a dedicated evidence-guided prompting strategy. By integrating model predictions, feature attribution results and neurophysiological evidence, the proposed framework guides a frozen LLM through evidence-guided inference to automatically generate coherent, evidence-based and neurophysiologically interpretable textual explanations.
4. Extensive experiments are conducted using multiple mainstream EEG encoding models to comprehensively evaluate the proposed evidence-guided inference framework across different network architectures. The experimental results demonstrate its generalizability, compatibility, and robustness, showing that the framework is encoder-agnostic and can be easily adapted to various EEG backbone models for practical deployment.

## 2 Materials and methods

### 2.1 Overall framework

The proposed E-MDD framework consists of three sequential stages: depression classification, evidence construction, and evidence-guided reasoning, as illustrated in Figure 1. Following a hierarchical processing pipeline, the framework progressively analyzes EEG signals from intelligent prediction to mechanism-oriented interpretation. Specifically, an EEG feature encoder is first employed to perform automated depression classification and generate quantitative prediction results. Based on these predictions, an interpretable evidence framework is constructed by integrating neurophysiological prior knowledge with model-derived evidence.Finally, a LLM generates evidence-guided short-text explanations, thereby establishing a complete analytical pipeline from automated detection to neurophysiological interpretation.

**Figure 1:**
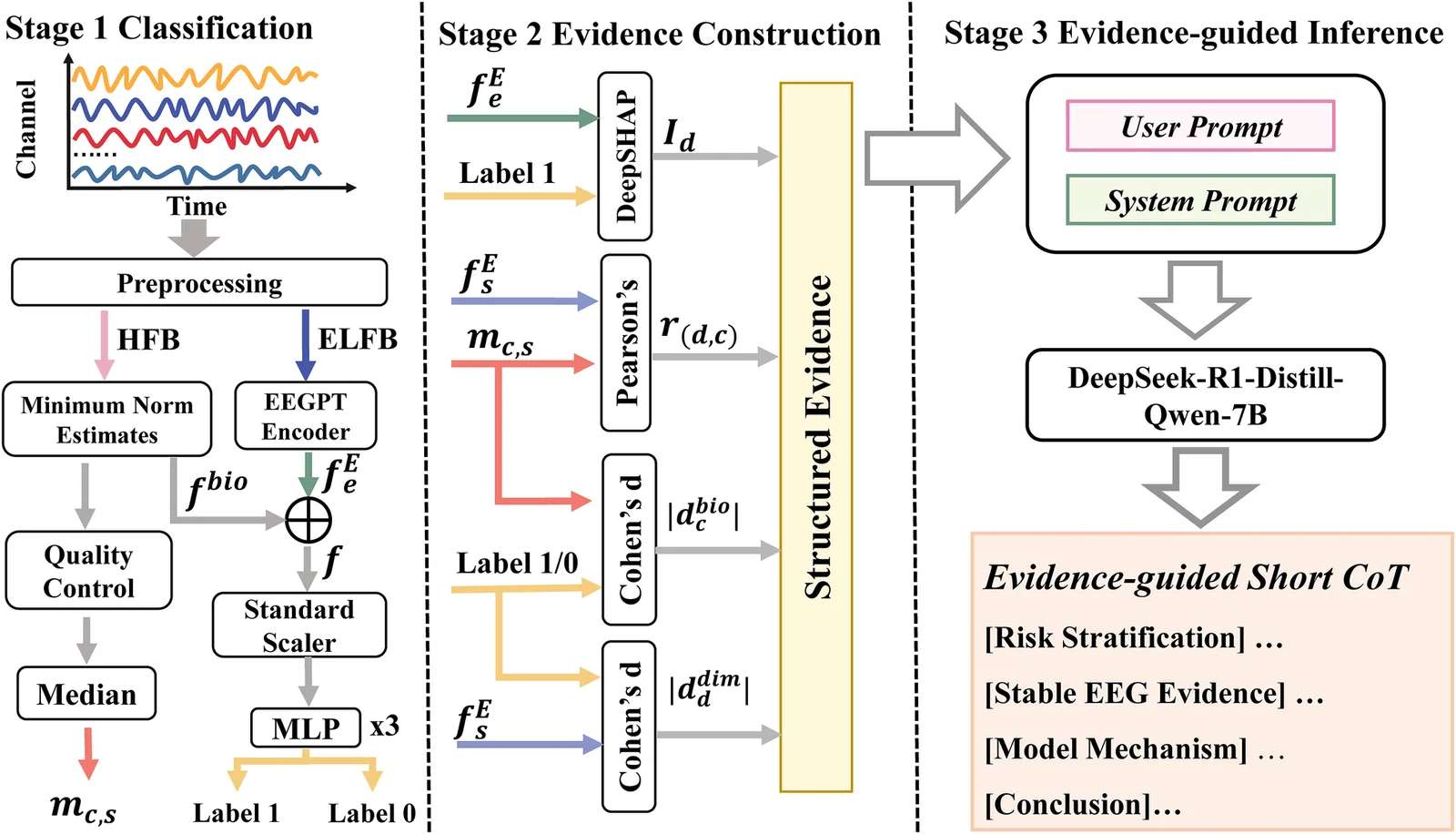
Overall architecture of the proposed E-MDD framework. The framework consists of three sequential stages. Stage 1 Classification: Preprocessed resting-state EEG signals are fed into a EEG latent feature branch (ELFB) and a handcrafted feature branch (HFB) to extract latent EEG representations and neurophysiological biomarkers, whose fused features are used for epoch-level and subject-level depression classification. Stage 2 Evidence Construction: Discriminative latent EEG dimensions are identified and aligned with conventional EEG biomarkers to construct structured evidence linking model predictions to neurophysiological mechanisms. Stage 3 Evidence-guided Inference: The structured evidence is provided to a frozen LLM through evidence-guided prompts, enabling the generation of concise, standardized, and neurophysiologically grounded textual explanations.

### 2.2 Classification stage

This section presents the classification stage of the proposed E-MDD framework to distinguish patients with depression from healthy controls (HC). This module adopts a dual-branch early feature fusion strategy, in which preprocessed EEG segments are simultaneously processed by two parallel feature extraction branches. The high-dimensional EEG feature branch (ELFB) generates a 512-dimensional latent EEG representation $f_{e}^{E}$, while the handcrafted feature branch (HFB) extracts neurophysiologically interpretable biomarkers $f^{\text{bio}}$. After feature extraction, the two heterogeneous feature representations are concatenated at the feature level to form a unified representation. The fused representation is subsequently fed into a lightweight classifier to generate prediction probabilities to distinguish between depression and healthy samples.

#### 2.2.1 EEG latent feature branch

First, the preprocessed EEG epochs are fed into the parameter-frozen EEGPT encoder (16, 21). The EEGPT encoder divides each EEG epoch into P spatio-temporal patches and outputs an embedding vector for each patch. Since different patch representations may capture complementary local temporal patterns, global average pooling is applied to aggregate all patch embeddings, resulting in a 512-dimensional epoch-level EEG latent feature representation $f_{e}^{E}$, as shown in Equation 1:

$$f_{e}^{E}=\frac{1}{P}\sum_{i=1}^{P}h_{i}\in R^{512}$$ (1)

where $h_{i}$ represents the embedding vector i -th corresponding to the patch i -th.

#### 2.2.2 Handcrafted feature branch

The handcrafted feature branch used Python-based Magnetoencephalography and Electroencephalography analysis tools to extract physiologically interpretable EEG biomarkers from the same EEG epochs used by the ELFB as handcrafted features. For each EEG epoch, 80 power features were obtained from the absolute and relative power across five frequency bands, namely delta, theta, alpha, beta, and gamma, calculated over the whole brain and seven predefined regions: frontal, central, temporal, parietal, occipital, left, and right. Additionally, 16 features were derived, including four frequency-band power ratios, mean homologous-electrode asymmetry across four frequency bands, whole-left–whole-right asymmetry across four frequency bands, three Hjorth parameters, and one spectral entropy feature. Collectively, these features resulted in an initial candidate biomarker set comprising 96 features.

Subsequently, epoch-level features from each subject in the development set were aggregated to the subject level, and the distributional differences between the MDD and HC were assessed for each handcrafted feature. Because some features may not satisfy the assumption of normality, a two-sided Mann–Whitney U test was employed to assess between-group differences and obtain the raw p values. The 96 candidate features were then ranked in ascending order of p value. Following sensitivity analysis, 20 handcraft features $f^{\text{bio}}$ were finally selected and concatenated with the 512-dimensional EEGPT latent representation to form the fused feature representation f, as shown in Equation 2:

$$f=\left[f_{e}^{E};\;m_{e}\right]\in R^{532}$$ (2)

Subsequently, the fused representation was standardized using StandardScaler and fed into the classification head for binary classification of MDD and HC.

#### 2.2.3 Classification strategy

The classification head consists of two hidden layers. The first hidden layer sequentially applies Batch Normalization, ReLU activation, and Dropout. The second hidden layer applies ReLU activation and Dropout without Batch Normalization. The final fully connected layer maps the learned representation to the classification output. The corresponding computation process is defined as shown in Equations 3–5:

$$z_{1}={\mathrm{Dropout}}_{0.45}\left(\mathrm{ReLU}\left(\mathrm{BN}\left(W_{1}f+b_{1}\right)\right)\right),\;W_{1}\in R^{256\times 532}$$ (3)

$$z_{2}={\mathrm{Dropout}}_{0.25}\left(\mathrm{ReLU}\left(W_{2}z_{1}+b_{2}\right)\right),\;W_{2}\in R^{64\times 256}$$ (4)

$$o=W_{3}z_{2}+b_{3},\;W_{3}\in R^{2\times 64}$$ (5)

The output $\mathrm{logits}(o=[o_{\mathrm{HC}},o_{\mathrm{MDD}}{]}^{⊤})$ are transformed into class probabilities using the Softmax function, where the probability corresponding to the MDD class is taken as the epoch-level MDD prediction probability $p_{e}$, as shown in Equation 6:

$$p_{e}=\frac{\exp(o_{\mathrm{MDD},e})}{\exp(o_{\mathrm{HC},e})+\exp(o_{\mathrm{MDD},e})}$$ (6)

The prediction probabilities of all EEG epochs belonging to the same subject are aggregated by averaging to obtain the subject-level MDD prediction probability $p_{s}$, as shown in Equation 7:

$$p_{s}=\frac{1}{N_{s}}\sum_{e\in E_{s}}p_{e}$$ (7)

where $E_{s}$ denotes the set of EEG epochs belonging to the subject s, and Ns represents the corresponding number of EEG epochs.

### 2.3 Evidence construction stage

The evidence construction stage aims to establish an evidence-guided explainable framework that bridges the high-dimensional latent representation of EEG $f_{e}^{E}$ and neurophysiological biomarkers. By identifying the precise correspondence between each latent dimension and its associated biomarkers, this stage generates stable, traceable, and auditable subject-level evidence for model explanation. This stage comprises four sequential components: SHapley Additive exPlanations (SHAP)-based feature attribution, cross-space semantic alignment (22), evidence quality control, and structured evidence construction. Through a multi-level progressive evidence interpretation and filtering mechanism, the framework enables the reliable transformation of implicit high-dimensional EEG latent representations into interpretable, verifiable, and traceable neurophysiological semantic evidence.

#### 2.3.1 SHAP-based feature attribution analysis

To quantify the contribution of the EEGPT latent representation to depression classification decisions, DeepSHAP was employed to perform feature attribution analysis on the fused MLP classifier. By calculating the contribution of each latent feature dimension to the prediction of the MDD class, key latent representation dimensions with substantial contributions to MDD discrimination were identified.

The SHAP background samples were drawn exclusively from the development-set subjects with an equal number of samples per subject, while the independent test set was not involved in determining the background set size. Using a fixed random seed of 42, four EEG epochs were randomly sampled without replacement from each of the 41 development-set subjects, yielding a total of 164 epoch-level samples. Full 532-dimensional SHAP values were computed for all EEG epochs in the background set, followed by extraction of the top 512 EEGPT latent dimensions.

Let $\phi_{e,d}$ denote the signed SHAP value of epoch e for latent dimension d. To avoid biasing the global ranking toward subjects with a larger number of epochs, SHAP values were first averaged across epochs within each subject and subsequently averaged across the 41 development-set subjects with equal weighting. The resulting global importance of latent dimension d was shown in Equation 8:

$$I_{d}=\frac{1}{S}\sum_{s=1}^{S}\left(\frac{1}{n_{s}}\sum_{e\in s}|\phi_{e,d}|\right)$$ (8)

and the signed direction is given in Equation 9:

$${\bar{\phi }}_{d}=\frac{1}{S}\sum_{s=1}^{S}\left(\frac{1}{n_{s}}\sum_{e\in s}\phi_{e,d}\right)$$ (9)

Where, S=41 denotes the number of development-set subjects, and $n_{s}$ denotes the number of epochs for subject s. $I_{d}$ represents a non-negative importance measure, whereas the sign of ${\bar{\phi }}_{d}$ indicates whether latent dimension d globally increases or decreases the MDD logit. The 17 independent test-set subjects were used only for post hoc interpretation and ranking stability assessment and were not involved in the calculation of $I_{d}$, latent-dimension selection, or subsequent representation alignment. After being computed and frozen on the development set, the subject-balanced importance $I_{d}$ was used in subsequent cross-space semantic alignment and as the model-importance component of the integrated priority score priority(d,c).

#### 2.3.2 Representation alignment

Establishing stable alignment relationships between the latent dimensions of $f_{e}^{E}$ and neurophysiological biomarkers provides interpretable evidence to understand the model decision process. Therefore, this study quantifies the correspondence between the latent dimensions of the EEG and neurophysiological biomarkers using Pearson correlation coefficients and the effects of the inter-group.

Since the cross-space alignment relationship is established at the subject level, the latent representations at the epoch-level of EEG $f_{e}^{E}$ are first aggregated into the representations at the subject level $f_{s}^{E}$, as shown in Equation 10:

$$f_{s}^{E}=\frac{1}{N_{s}}\sum_{e\in E_{s}}f_{e}^{E}$$ (10)

Neurophysiological biomarkers at the subject-level are represented by the median values across all EEG epochs of each subject, as shown in Equation 11.

$$m_{c,s}=\text{median}\left(\{m_{c,e}\;:\;e\in E_{s}\}\right)$$ (11)

denotes the value of neurophysiological biomarker c extracted from the EEG epoch e.

For the d-th EEGPT latent dimension and the c-th biomarker, the Pearson correlation coefficient $r_{d,c}$ was calculated to quantify their linear association. Statistical significance was assessed using a two-sided test. Specifically, the correlation coefficient was first transformed into a t-statistic, as shown in Equation 12:

$$t_{\left(d,c\right)}=r_{\left(d,c\right)}\sqrt{\frac{n-2}{1-r_{\left(d,c\right)}^{2}}},\;\mathrm{df}=n-2$$ (12)

The corresponding raw two-sided p-value was then calculated as shown in Equation 13:

$$p_{\left(d,c\right)}=2\left[1-F_{t,n-2}\left(|t_{\left(d,c\right)}|\right)\right]$$ (13)

Where, $F_{t,n-2}$ denotes the cumulative distribution function of the Student’s t-distribution with n−2 degrees of freedom.

A total of 512×8=4096 latent dimension–biomarker association tests were performed. The raw p values from all 4,096 two-sided Pearson correlation tests were jointly corrected using the Benjamini–Hochberg false discovery rate (BH-FDR) procedure, and the corresponding adjusted q values were reported. Pairs satisfying q<0.05 were retained for subsequent integrated priority scoring and construction of the mapping pool.

We further calculated Cohen’s effect sizes between each latent dimension of the EEG in $f_{s}^{E}$, each neurophysiological biomarker $m_{(}c,s)$, and the subject label $y_{s}\in \{0,1\}$, respectively (23). These effect sizes were used to quantify the inherent discriminative capacity of latent dimensions and biomarkers, and were indicated as $\left|d_{d}^{dim}\right|$ and $\left|d_{c}^{bio}\right|$, respectively.

Finally, $I_{d}$, $\left|r_{\left(d,c\right)}\right|$, $\left|d_{d}^{\dim}\right|$, and $\left|d_{c}^{\mathrm{bio}}\right|$ were normalized using Min–Max normalization. To quantitatively integrate the complementary information provided by latent-dimension decision importance, latent dimension–biomarker correlation strength, biomarker effect size, and latent-dimension effect size, the integrated priority score was defined as a weighted linear combination of the four normalized components, as shown in Equation 14:

$$\mathrm{Priority}(d,c)=\alpha {\tilde{I}}_{d}+\beta \tilde{\left|r_{\left(d,c\right)}\right|}+\gamma \tilde{\left|d_{c}^{\mathrm{bio}}\right|}+\delta \tilde{\left|d_{d}^{\dim}\right|}$$ (14)

Where, ${\tilde{I}}_{d}$, $\tilde{\left|r_{\left(d,c\right)}\right|}$, $\tilde{\left|d_{c}^{bio}\right|}$, and $\tilde{\left|d_{d}^{dim}\right|}$ denote the corresponding values after Min–Max normalization. The weights were set to α=0.35, β=0.30, γ=0.20, and δ=0.15, respectively, with the weights summing to 1.

To prevent frequently occurring individual biomarkers from dominating the mapping pool, 20 dim–bio mappings were constructed. For each biomarker, the two significant pairs with the highest priority scores were preferentially retained. If fewer than two significant pairs were available for a given biomarker, all significant pairs were retained. Subsequently, the 20 mappings were completed by ranking the remaining pairs in descending order of Priority, subject to a maximum of three pairs per biomarker.

#### 2.3.3 Epoch-level quality control

To mitigate explanation instability and semantic deviations caused by noisy EEG epochs, an epoch-level quality control (QC) mechanism was introduced into the handcrafted feature branch. Based on robust z-score statistics, the proposed QC strategy performs anomaly detection on the total power, band-specific energy, and spectral characteristics of each EEG epoch, enabling the identification of typical abnormal patterns, including global power abnormalities, excessive high-frequency energy elevation, low-frequency signal drift, missing features, and incomplete data. When an EEG epoch meets any abnormality criterion, it is flagged as an invalid sample and excluded from subsequent structured evidence construction and explanation generation. This procedure ensures the reliability and accuracy of the final evidence used for the interpretation of the model.

### 2.4 Evidence-guided inference

This study designs an evidence-guided short-text explanation generation framework to transform the decision basis of deep learning-based EEG classification into structured textual explanations with high readability and logical consistency (24–26). Specifically, the high-quality structured evidence constructed in Stage II is used as the core input, combined with customized evidence-guided prompts and inference constraint rules. A parameter-frozen large language model, DeepSeek-R1-Distill-Qwen-7B, is used to perform constrained reasoning and ultimately generate concise, compliant and physiologically interpretable short-text explanations for depression classification.

As shown in Figure 2, the System Prompt is designed to define the role of the model, the boundaries of tasks, and the generation constraints. It instructs the model to generate explanations solely based on the provided evidence, without revealing its internal reasoning process, referring to ground-truth labels, dataset labels, or subject identity information, or producing diagnostic conclusions beyond the available evidence. These constraints effectively prevent label leakage and prompt contamination.

**Figure 2:**
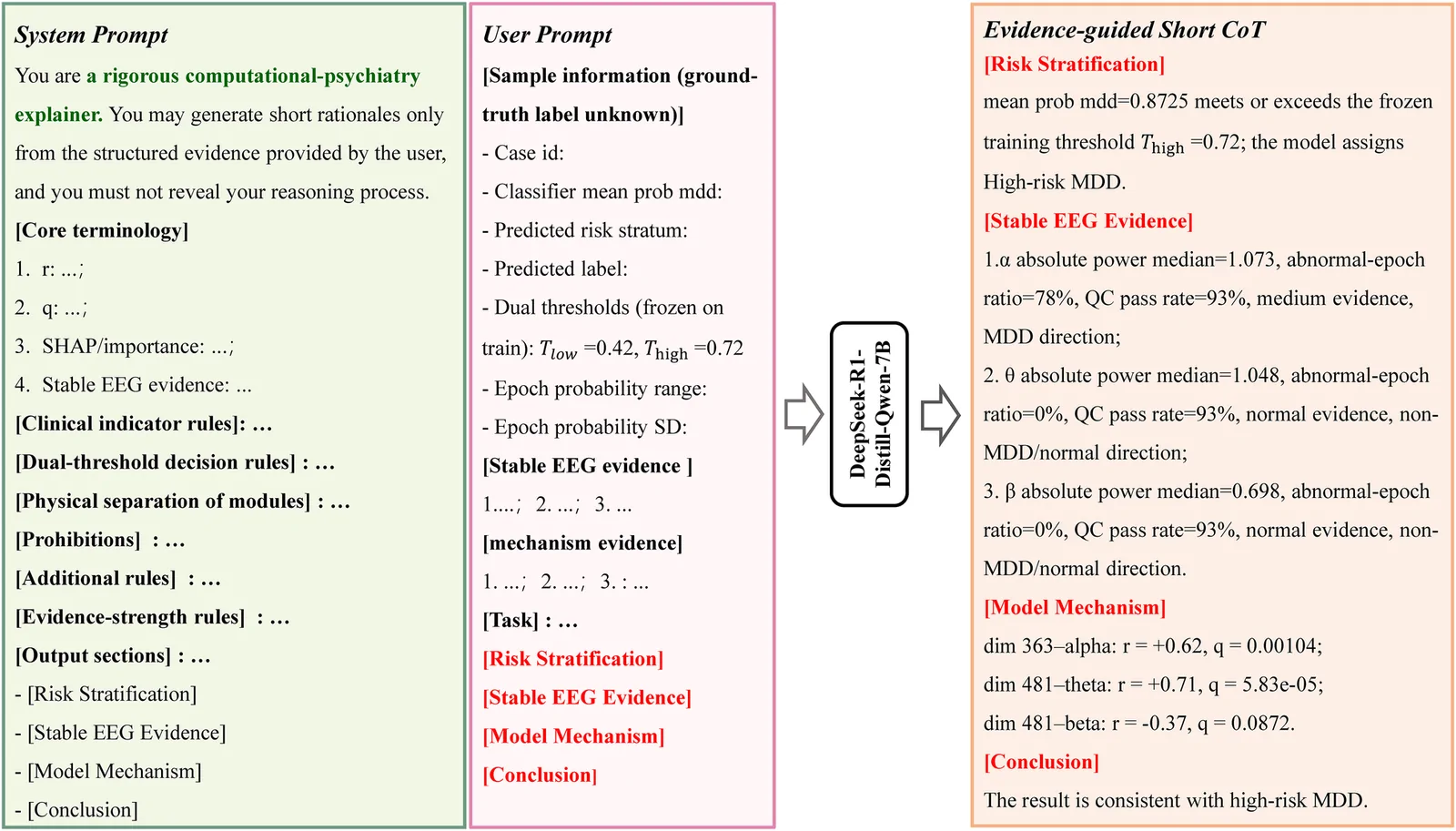
The structure and detailed contents of the system prompt and user prompt, together with an example of the evidence-guided short CoT explanation generated by the proposed prompting strategy.

The User Prompt provides anonymized sample information, subject-level prediction probabilities, risk stratification results, frozen thresholds, and precomputed stable EEG evidence and mechanistic evidence. Stable evidence is obtained after quality control and priority-based selection during the training stage. A maximum of three evidence entries are retained for each subject, and the strength of the evidence is determined based on the pass rate of the QC and the proportion of abnormal epochs. The model is only allowed to reference valid evidence and is instructed to prioritize high-confidence evidence consistent with the current risk stratification when generating explanations.

The prompt requires the generated explanation to strictly follow a four-part structure: Risk Stratification, Stable EEG Evidence, Model Mechanism, and Conclusion. It also restricts the output length and format while prohibiting the generation of chain-of-thought labels, code blocks, or any content unrelated to the explanation. In addition, an automatic validation process is applied to check the output generated in terms of format compliance, length constraints, and sensitive information leakage. If any constraint is violated, the system returns to a deterministic template-based generation strategy using the same evidence package, ensuring the stability and reproducibility of the generated explanations.

In summary, the proposed evidence-guided prompt strictly constrains the functional scope of the large language model by integrating role constraints, frozen inference rules, structured evidence injection, knowledge constraints, and a standardized output template. Under these constraints, the LLM is restricted to faithful, controllable, and traceable natural-language translation of the evidence underlying the model’s decisions, thereby mitigating bias and hallucinations arising from autonomous reasoning. This provides a unified and standardized set of rule-based constraints for subsequent explanation quality assessment and faithfulness validation.

## 3 Experiments

### 3.1 Dataset and EEG preprocessing

This study utilized the publicly available EEG dataset for depression collected by Mumtaz et al. (27). Participants were recruited from the psychiatric outpatient clinic of Hospital Universiti Sains Malaysia (HUSM). The study protocol was approved by the institutional ethics committee of HUSM, and written informed consent was obtained from all participants. MDD was diagnosed by experienced psychiatrists according to the Diagnostic and Statistical Manual of Mental Disorders, Fourth Edition (DSM-IV) criteria. Participants with psychotic symptoms, pregnancy, alcohol dependence, smoking, or epilepsy were excluded. HCs underwent psychiatric and physical screening to confirm the absence of relevant disorders. Depression severity was assessed using the Beck Depression Inventory-II (BDI-II) and the Hospital Anxiety and Depression Scale (HADS). Patients with MDD underwent a 2-week medication washout period before their first EEG recording, after which they received selective serotonin reuptake inhibitor (SSRI) antidepressant treatment under medical supervision. After subject screening, resting-state, eyes-closed EEG recordings were obtained from 34 MDD patients and 30 HC. Six subjects from the original dataset were excluded due to missing complete EEG recordings; consequently, only 58 subjects’ EEG data were available for the present study. In the original report of this cohort, the MDD group had a mean age of 40.33±12.86 years and comprised 16 males and 17 females. The healthy control group consisted of age-matched participants, with a mean age of 38.23±15.64 years.

The EEG recordings consisted of 19 channels following the international 10–20 system, with a sampling rate of 256 Hz and ear-referenced montage. The amplifier incorporated a 0.5–70 Hz band-pass filter and a 50 Hz notch filter. Resting-state EEG recordings were acquired under both eyes-closed and eyes-open conditions, with each recording lasting approximately 5 min. Only the eyes-closed resting-state EEG data were used in this study. Building upon the filters applied during acquisition, the raw EEG signals were further processed using a 50 Hz notch filter and a 0.1–70 Hz band-pass filter, followed by independent component analysis (ICA) to remove ocular and muscle artifacts (8, 28). The continuous EEG signals were then segmented using a 6-s sliding window with 50% overlap, followed by extraction of the central 4 s from each window. Each resulting epoch comprised 19 channels with 1,024 sampling points per channel, corresponding to 4 s × 256 Hz. After preprocessing, a total of 5,721 EEG epochs were obtained. The independent test set comprised 17 subjects, including 9 patients with MDD and 8 healthy controls, yielding a total of 1,702 EEG epochs.

### 3.2 Data partitioning and data leakage control

All procedures involving model parameter optimization, feature selection, statistical parameter estimation, decision-rule determination, and explanation-rule construction were strictly confined to the development set. The independent test set was locked immediately after data partitioning and was used exclusively for final performance evaluation and post hoc interpretation based on the prespecified frozen rules.

The data were first partitioned at the subject level into development and independent test sets using a 7:3 ratio, ensuring that all EEG epochs from the same subject were assigned exclusively to one set. This partition yielded a development set of 41 subjects and an independent test set of 17 subjects. Model development was conducted exclusively within the development set. Five-fold stratified group cross-validation was performed using StratifiedGroupKFold, with subject identity serving as the grouping variable and diagnostic labels as the stratification criterion. In each fold, approximately 80% of the development-set subjects were used for training and the remaining 20% for validation. All EEG epochs from a given subject were consistently assigned to the same partition, thereby preventing subject-level information leakage caused by random epoch-level splitting. The random seeds for NumPy, PyTorch, and data partitioning were all fixed at 42.

During the classification stage, the publicly available pretrained EEGPT model was employed as the EEG encoder (16). The encoder parameters were kept frozen throughout the entire training process, and only the classification head was optimized for MDD detection. During inference, the parameters of the large language model were likewise kept frozen, reducing computational overhead while preserving the general knowledge and reasoning capabilities acquired by the pretrained language model.

In the sensitivity analysis of the number of handcrafted EEG features, the Mann–Whitney *U* test–based feature ranking, median estimation for missing-value imputation, and standardization parameter fitting were performed independently using only the training subjects within each fold. The resulting parameters were then applied to the validation subjects in the corresponding fold. After the final 20-dimensional handcrafted feature set was determined and frozen based on the entire development set, it was subsequently applied to the independent test set.

Dimension–biomarker representation alignment was likewise performed exclusively within the development set. Following biomarker quality control, 35 development-set subjects were included in the Pearson correlation analysis, BH-FDR correction, and Cohen’s d effect-size calculation. The resulting 1,140 significant pairs, Priority rankings, and the 20-pair balanced mapping pool were all frozen before evaluation on the independent test set. The independent test subjects were used only with the frozen model, feature set, standardization parameters, and mapping pool to generate predictions and explanations.

The 95% confidence intervals for model performance were estimated using subject-clustered bootstrap resampling. In each iteration, subjects were sampled with replacement as the resampling unit, while all EEG epochs belonging to each selected subject were retained to preserve the within-subject dependence structure. This procedure was repeated 2,000 times, and the 2.5th and 97.5th percentiles of the resulting bootstrap distributions were taken as the 95% confidence interval. Finally, the independent test set was used exclusively for one-time evaluation of the frozen model in terms of ACC, F1, AUC, SEN, and SPE, as well as for post hoc interpretation based on the frozen evidence mapping, and did not contribute to the selection of any model or explanation rules.

### 3.3 Evaluation metrics

The evaluation metrics for classification performance included accuracy (ACC), precision, recall, F1-score, SENitivity (SEN), specificity (SPE) and the area under the receiver operating characteristic curve (AUC).

Following established approaches for evaluating LLM-generated text (26, 29, 30), the evidence-guided short-text explanations were evaluated from two complementary dimensions: automated quality compliance and evidence faithfulness (31, 32).

The automatic quality compliance evaluation consisted of five rule-based metrics, which were designed to assess whether the generated explanations satisfied the predefined structural, linguistic, and safety constraints. Specifically, (1) Structural Completeness evaluates whether the generated explanation fully contains the predefined reasoning structure; (2) Template Compliance assesses whether the output conforms to the unified explanation template; (3) Terminology Consistency verifies whether medical terminology and EEG-related expressions are standardized and consistently used; (4) Text Readability examines whether the generated text satisfies predefined sentence-level readability rules, including complete sentence structures, fluent expressions, and the absence of redundant or abnormal content (29); and (5) No Information Leak detects whether the explanation contains sensitive information, such as ground-truth labels, subject identity information, or prompt templates (30, 31).

All five metrics were automatically determined according to predefined rules, and the resulting score summarizes the degree of compliance with the predefined explanation-generation protocol.

The faithfulness evaluation was designed to assess whether the generated explanations remained grounded in the evidence underlying the model’s decisions (31–33). It consisted of five metrics with their specific definitions as follows: (1) Decision Alignment verifies whether the risk level described in the explanation is consistent with the prediction outcome of the classification model (34); (2) Feature Alignment assesses whether the explanation accurately references the key high-dimensional EEG features identified by the model (35); (3) Biomarker Alignment evaluates whether the biomarkers mentioned in the generated explanation are consistent with the case-specific biomarkers provided as structured input evidence, including their corresponding biomarker names and values (34, 35); (4) The reliability of the direction of the evidence determines whether the direction of the EEG alterations described in the explanation agrees with the input evidence provided; and (5) the traceability of the mechanism verifies whether each explanation can be fully traced back to the prediction of the model, the dimensions of the corresponding important latent characteristic, and their associated neurophysiological indicators, thus establishing a complete and continuous evidence chain (32, 33).

### 3.4 Implementation details

All experiments were implemented in Python and conducted on an NVIDIA GPU platform. Deep learning models were developed using the PyTorch framework. EEG signal preprocessing and spectral analysis were performed with MNE-Python, while model training, group-based cross-validation, and performance evaluation were implemented using Scikit-learn. Feature attribution analysis was conducted using the SHAP library. The EEGNet and EEGConformer models were implemented based on the Braindecode toolkit, and the large language model inference was performed using the Hugging Face Transformers and BitsAndBytes frameworks.

### 3.5 Validation strategy

To comprehensively evaluate the effectiveness and generalizability of the proposed explainable framework, three representative EEG encoding models were selected as feature extraction backbones, including EEGPT, EEGNet, and EEGConformer. These three models correspond to the main technical paradigms in the field of EEG analysis, namely the pre-trained foundation model, the lightweight convolutional neural network, and the CNN–Transformer hybrid architecture, respectively. Together, they provide comprehensive coverage of various feature learning mechanisms. To ensure the fairness and validity of the comparative experiments, a unified experimental configuration and processing pipeline were adopted. Only the EEG encoder module was replaced in different models, while all other network architectures, training strategies, and inference parameters were kept exactly the same. The core purpose of this experiment is to verify the adaptability and operational stability of the proposed framework across various deep learning-based EEG models. If multiple heterogeneous encoders can achieve stable mapping of neurophysiological features within a unified framework and consistently yield reliable classification results and interpretable evidence, it can demonstrate that the proposed framework possesses model-agnostic properties and cross-architecture generalization ability.

## 4 Results

### 4.1 Evidence-guided short-text explanation

An example of the evidence-guided short-text explanation generated by the proposed explainable framework is presented in Figure 3.

**Figure 3:**
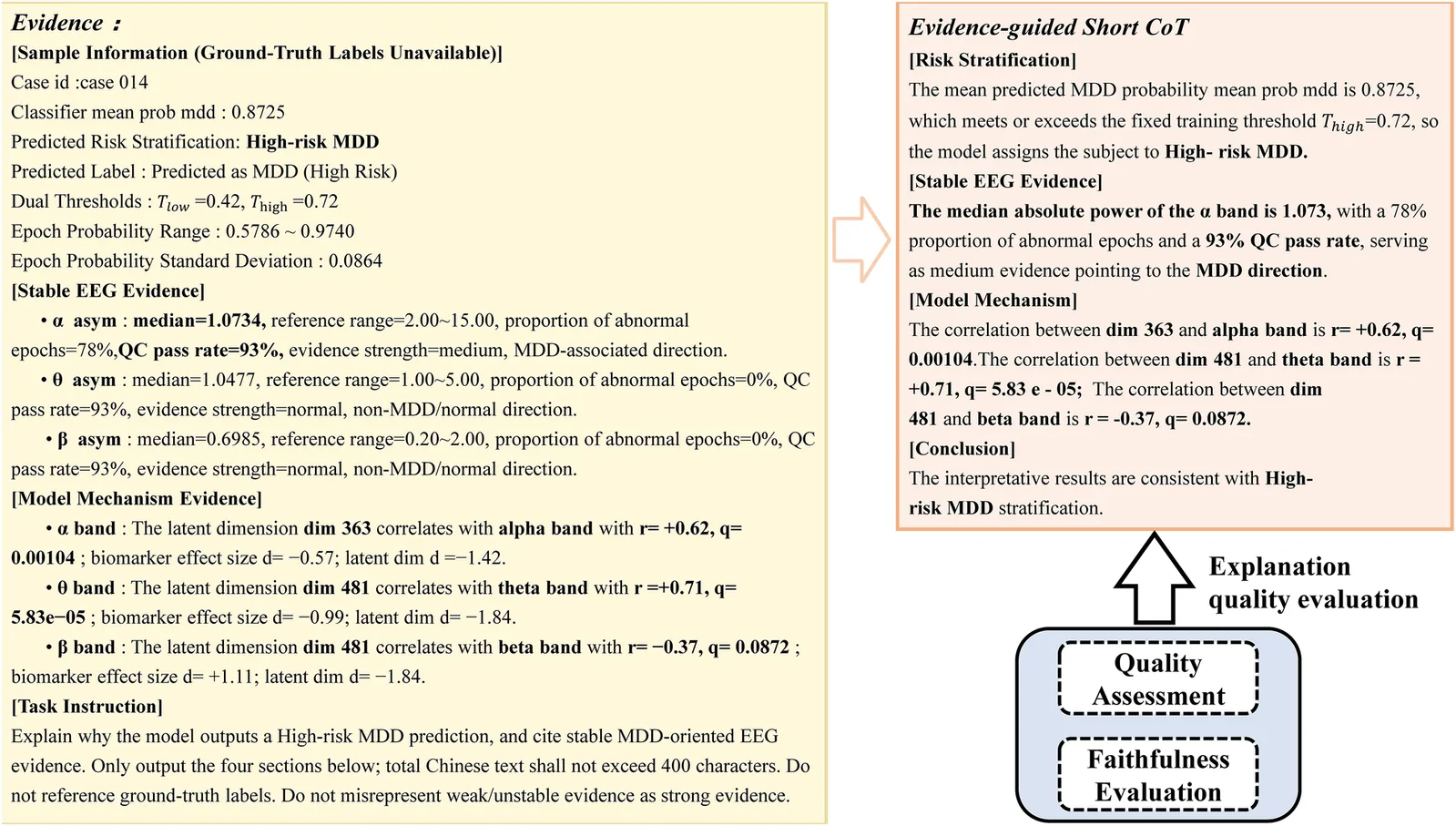
Examples of structured evidence construction and short explanation generation in the proposed explainable framework.

As shown in Figure 3, the short-text explanation generated by the proposed framework provides a comprehensive description of the outcome of the prediction of the model, the underlying neurophysiological evidence, and the corresponding EEG features. The entire explanation process is based solely on the provided input evidence, without introducing additional diagnostic information or unsupported inferences.

As shown in Table 1, all generated explanations achieved a score of 100.0 across all five automated quality compliance metrics. These results demonstrate that the evidence-guided short-text explanations consistently satisfied the predefined constraints on structure, template adherence, terminology consistency, readability, and information leakage prevention.

**Table 1:** Automated quality compliance and faithfulness evaluation scores.

Category	Metrics	Score
Automated quality compliance scores	Structure completeness	100
	Template compliance	100
	Terminology consistency	100
	Text readability	100
	No information leakage	100
	Overall	100
Faithfulness evaluation scores	Decision alignment	100
	Feature alignment	79.4
	Biomarker alignment	100
	Evidence direction	100
	Mechanism traceability	95.9
	Overall faithfulness	95.5

The proposed explanation generation framework achieved high evidence faithfulness, with an overall faithfulness evaluation score of 95.5. Specifically, Decision Alignment, Biomarker Alignment, and Evidence Direction all reached 100.0, indicating that the generated explanations remained consistent with the model prediction, the corresponding neurophysiological biomarkers, and the direction of the provided EEG evidence. The Mechanism Traceability score reached 95.9, indicating that most explanations could be traced from the model prediction through the corresponding latent dimensions to their associated neurophysiological indicators. The relatively lower Feature Alignment score of 79.4 was mainly attributed to the selective use of representative evidence rather than exhaustive coverage of all identified key features. Thus, the faithfulness results provide evidence that the generated explanations were grounded in the model-derived evidence, whereas the 100.0 automatic quality-compliance score should be interpreted only as successful adherence to the predefined generation protocol.

### 4.2 Latent dimension–biomarker associations

As shown in Table 2, all the 20 pairs of latent dimensions—biomarkers of EEGPT remained significant after FDR correction (q<0.05). The identified associations spanned spectral power features across the δ, θ, α, β, and γ frequency bands, as well as delta asymmetry, theta asymmetry, and alpha asymmetry measures. Significantly, individual latent dimensions can establish associations with multiple physiological biomarkers, revealing a one-to-many correspondence pattern. The maximum correlation coefficient reaches r=0.87. These findings demonstrate that the latent representations learned by EEGPT encode information pertaining to both localized spectral characteristics and hemispheric asymmetry, which lays the foundation for building an EEGPT dimension–biomarker mapping dictionary in subsequent representation alignment analyses.

**Table 2:** EEGPT Top-20 latent dimension–biomarker Pearson correlations on the development set.

Rank	Dim.	Biomarker	r	p	q	95% CI
1	219	θ	0.8737	$7.38\times 10^{-12}$	$1.51\times 10^{-8}$	(0.7624,0.9348)
2	259	θ	0.7293	$6.73\times 10^{-7}$	$2.39\times 10^{-5}$	(0.5232,0.8548)
3	371	θ	0.7624	$1.02\times 10^{-7}$	$5.90\times 10^{-6}$	(0.5754,0.8737)
4	259	γ	0.6271	$5.54\times 10^{-5}$	$8.00\times 10^{-4}$	(0.3715,0.7944)
5	455	β	0.6513	$2.26\times 10^{-5}$	$4.00\times 10^{-4}$	(0.4062,0.8090)
6	248	β	0.4438	0.0076	0.0316	(0.1298,0.6770)
7	483	β	−0.5021	0.0021	0.0125	(−0.7156,−0.2028)
8	448	α	−0.6110	$9.69\times 10^{-5}$	0.0012	(−0.7845,−0.3487)
9	219	γ	0.6449	$2.89\times 10^{-5}$	$5.00\times 10^{-4}$	(0.3970,0.8051)
10	363	δ	0.5048	0.0020	0.0120	(0.2062,0.7174)
11	407	α	−0.6542	$2.02\times 10^{-5}$	$3.00\times 10^{-4}$	(−0.8107,−0.4105)
12	363	α	0.6177	$7.71\times 10^{-5}$	0.0010	(0.3582,0.7886)
13	018	γ	0.6955	$3.52\times 10^{-6}$	$8.43\times 10^{-5}$	(0.4716,0.8352)
14	269	δ	0.4372	0.0086	0.0350	(0.1217,0.6725)
15	160	δ	0.6137	$8.85\times 10^{-5}$	0.0011	(0.3525,0.7861)
16	248	$\alpha_{\mathrm{asym}}$	−0.4611	0.0053	0.0241	(−0.6885,−0.1511)
17	259	$\alpha_{\mathrm{asym}}$	0.4337	0.0092	0.0368	(0.1175,0.6701)
18	470	$\theta_{\mathrm{asym}}$	0.5212	0.0013	0.0090	(0.2274,0.7280)
19	176	$\theta_{\mathrm{asym}}$	0.5076	0.0019	0.0115	(0.2099,0.7192)
20	038	$\delta_{\mathrm{asym}}$	0.4289	0.0102	0.0395	(0.1116,0.6668)

## 5 Discussion

### 5.1 Cross-model classification performance

To systematically validate the effectiveness and generalizability of the proposed classification module, multiple comparative experiments were conducted by replacing the encoding backbone of the EEG feature. Specifically, EEGNet (36) and EEGConformer (37) were introduced as alternative models to extract EEG features. Additionally, these three models were evaluated using ten-fold cross-validation and leave-one-subject-out cross-validation (LOSO). Both experiments were conducted under consistent experimental conditions, including identical data partitioning strategies, classification training procedures, and evaluation protocols, to comprehensively assess the adaptability and generalization performance of the proposed framework across different EEG representation models.

The classification performance of the three EEG models is presented in the 5-fold results in Table 3. At the epoch level, all three encoders achieved stable classification performance, with AUC values exceeding 0.82. These results demonstrate that the proposed classification pipeline can effectively leverage diverse deep learning-based EEG representations for depression detection. The EEGPT-based approach achieved a ACC of 75.32% and an AUC of 0.8273 on the held-out test set, indicating a favorable discriminative capacity for distinguishing MDD from HC. At the subject level, aggregation of prediction probabilities across multiple EEG epochs from the same subject yielded a further improvement in classification performance across all three encoders. Specifically, the EEGPT-based framework achieved an accuracy of 82.35%, a sensitivity of 88.89%, and an F1 score of 84.21%, whereas EEGNet attained the highest subject-level AUC of 0.9444. The observed performance differences among the three encoders may be attributable primarily to differences in their underlying EEG representation learning strategies. The receiver operating characteristic (ROC) curves of the different EEG encoders at both the epoch and subject levels, shown in Figure 4, further support the stable discriminative performance of the evaluated models.

**Table 3:** Epoch-level and subject-level classification comparison of three models.

Level	Validation	Model	ACC (95% CI)	F1 (95% CI)	AUC (95% CI)	SEN (95% CI)	SPE (95% CI)
Epoch-level	Held-out test	EEGNet	0.7673 (0.5965, 0.8970)	0.7335 (0.5055, 0.8817)	0.8975 (0.7379, 0.9875)	0.6279 (0.3832, 0.8476)	0.9125 (0.7781, 1.0000)
		EEGConformer	0.8043 (0.6232, 0.9449)	0.8019 (0.5552, 0.9493)	0.8842 (0.7248, 0.9881)	0.7765 (0.5109, 0.9929)	0.8333 (0.5607, 1.0000)
		EEGPT	0.7532 (0.5956, 0.8848)	0.7578 (0.5381, 0.8910)	0.8273 (0.6251, 0.9627)	0.7569 (0.5338, 0.9203)	0.7494 (0.4846, 0.9904)
	10-fold CV (N=58)	EEGNet	0.8132 (0.6437, 0.9482)	0.8143 (0.5988, 0.9486)	0.9009 (0.7476, 0.9892)	0.8030 (0.5696, 0.9869)	0.8237 (0.5658, 1.0000)
		EEGConformer	0.8067 (0.6289, 0.9426)	0.8054 (0.5668, 0.9504)	0.8919 (0.7370, 0.9942)	0.7846 (0.5272, 0.9946)	0.8297 (0.5607, 0.9946)
		EEGPT	0.6821 (0.5000, 0.8296)	0.6122 (0.3807, 0.7893)	0.8358 (0.6275, 0.9770)	0.4919 (0.2815, 0.7157)	0.8801 (0.6978, 0.9981)
	LOSO (N=58)	EEGNet	0.7967 (0.6112, 0.9424)	0.7984 (0.5624, 0.9447)	0.8927 (0.7286, 0.9858)	0.7892 (0.5359, 0.9869)	0.8046 (0.5215, 1.0000)
		EEGConformer	0.8032 (0.6237, 0.9418)	0.8031 (0.5645, 0.9501)	0.8869 (0.7280, 0.9945)	0.7869 (0.5298, 0.9955)	0.8201 (0.5437, 0.9941)
		EEGPT	0.6904 (0.5122, 0.8362)	0.6446 (0.4036, 0.8152)	0.8063 (0.5773, 0.9625)	0.5507 (0.3253, 0.7793)	0.8357 (0.5994, 0.9975)
Subject-level	Held-out test	EEGNet	0.7647 (0.5294, 0.9412)	0.7143 (0.3333, 0.9333)	0.9444 (0.8182, 1.0000)	0.5556 (0.2000, 0.8750)	1.0000 (1.0000, 1.0000)
		EEGConformer	0.8235 (0.6471, 1.0000)	0.8235 (0.5714, 1.0000)	0.9444 (0.8182, 1.0000)	0.7778 (0.5000, 1.0000)	0.8750 (0.6000, 1.0000)
		EEGPT	0.8235 (0.6471, 1.0000)	0.8421 (0.6248, 1.0000)	0.8472 (0.6142, 1.0000)	0.8889 (0.6667, 1.0000)	0.7500 (0.4000, 1.0000)
	10-fold CV (N=58)	EEGNet	0.9483 (0.8793, 1.0000)	0.9492 (0.8800, 1.0000)	0.9810 (0.9467, 1.0000)	0.9333 (0.8333, 1.0000)	0.9643 (0.8788, 1.0000)
		EEGConformer	0.9310 (0.8621, 0.9828)	0.9333 (0.8571, 0.9855)	0.9524 (0.8833, 0.9975)	0.9333 (0.8333, 1.0000)	0.9286 (0.8182, 1.0000)
		EEGPT	0.8103 (0.7069, 0.8966)	0.8197 (0.6939, 0.9118)	0.9524 (0.8930, 0.9953)	0.8333 (0.6857, 0.9615)	0.7857 (0.6207, 0.9286)
	LOSO (N=58)	EEGNet	0.9138 (0.8276, 0.9828)	0.9180 (0.8302, 0.9811)	0.9833 (0.9524, 1.0000)	0.9333 (0.8333, 1.0000)	0.8929 (0.7667, 1.0000)
		EEGConformer	0.9310 (0.8621, 0.9828)	0.9333 (0.8571, 0.9855)	0.9643 (0.9119, 1.0000)	0.9333 (0.8333, 1.0000)	0.9286 (0.8182, 1.0000)
		EEGPT	0.9138 (0.8276, 0.9828)	0.9206 (0.8364, 0.9818)	0.9548 (0.8976, 0.9939)	0.9667 (0.8929, 1.0000)	0.8571 (0.7200, 0.9677)

**Figure 4:**
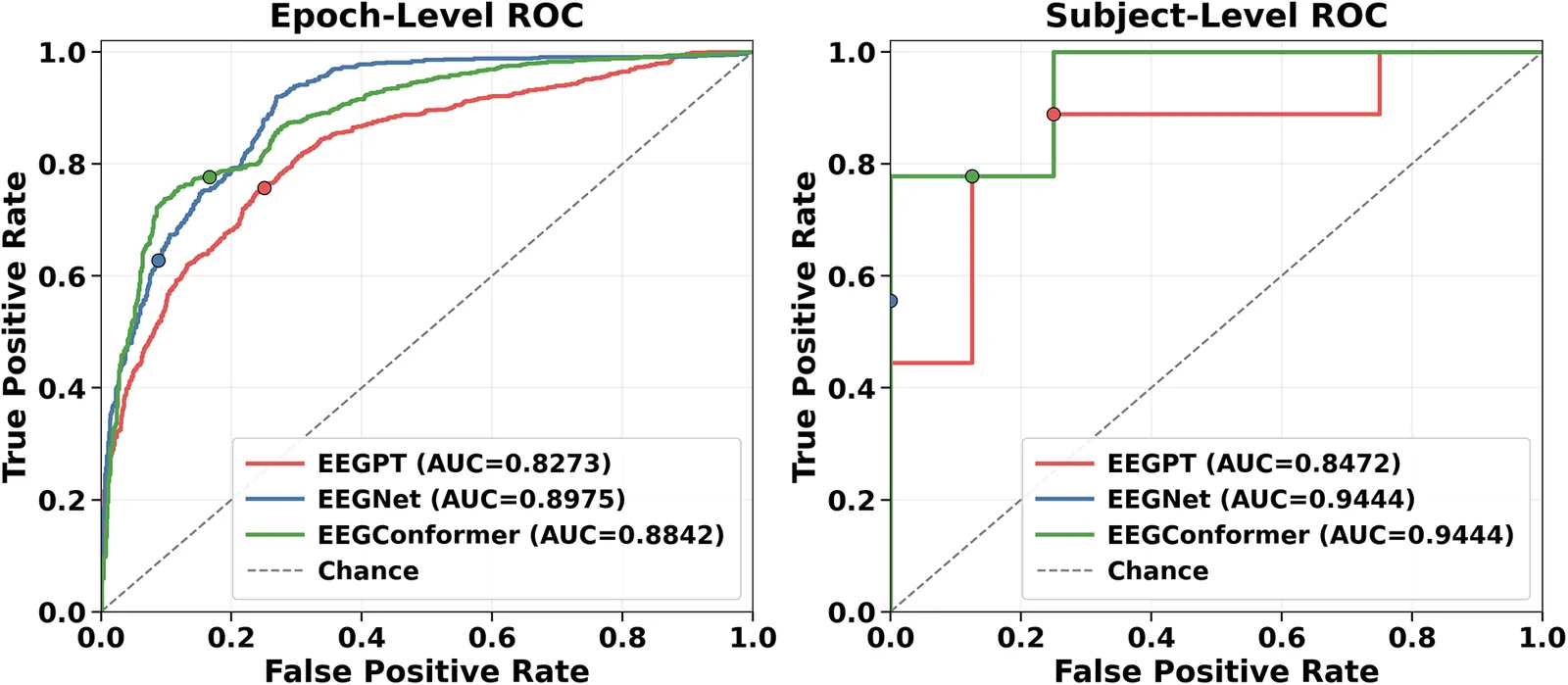
Receiver operating characteristic (ROC) curves of different EEG encoders for epoch-level and subject-level classification tasks.

To further assess the robustness of the classification model, additional ten-fold cross-validation and LOSO experiments were conducted. The corresponding results are reported under the 10-fold and LOSO settings in the Table 3. In the ten-fold cross-validation, the classification model maintained favorable generalization performance across different subject partitions, while exhibiting a relatively balanced trade-off between sensitivity and specificity. This suggests that the learned representations were not unduly dependent on a particular subset of subjects. Under the LOSO protocol, the model retained effective discriminative capability for previously held-out individuals, indicating that its classification performance was not solely attributable to a specific partitioning scheme. Taken together, the results from both evaluation protocols provide additional evidence supporting the robustness of the primary classification results under subject-independent evaluation.

Overall, under a unified experimental configuration, the three EEG encoders maintained relatively stable classification performance across multiple subject-independent evaluation protocols, supporting the adaptability and robustness of the proposed classification framework. Among the evaluated encoders, EEGPT not only achieved reliable depression detection performance across different validation settings but also provided high-dimensional EEG representations, thereby establishing a basis for subsequent neurophysiological representation alignment, analysis of important latent dimensions, and evidence-guided explanation generation.

### 5.2 Sensitivity analysis of handcrafted features

To determine the appropriate number of handcrafted biomarkers for the fused classification model, a sensitivity analysis was conducted on the development set with K∈{10,20,30,40,50,60,70,80,96}. In each fold, the handcrafted features were ranked by their Mann–Whitney p-values using only the training subjects, and the top-K features were selected. Classification performance and feature-selection stability were then evaluated using subject-grouped five-fold out-of-fold (OOF) predictions. As shown in Table 4, the subject-level AUC was highest at K=20, followed by K=10. As the number of selected features increased beyond 20, the performance metrics exhibited a fluctuating downward trend, with AUC decreasing to 0.9714 at K=60 and reaching 0.9786 when all 96 features were included. Feature-selection stability was relatively low at K=10, with a cross-fold Jaccard similarity of only 0.48, whereas the Jaccard similarity increased to 0.78 at K=20, with 15 features consistently selected across all five folds. These findings indicate that the classification benefit was largely saturated at K=20, and further increasing the number of selected features did not yield additional performance gains. Therefore, K=20 was selected for subsequent analyses.

**Table 4:** Handcrafted Top-K sensitivity on the development set.

K	Epoch-level			Subject-level		
	ACC	F1	AUC	ACC	F1	AUC
10	0.8771	0.8823	0.9612	0.9268	0.9333	0.9881
20	0.9035	0.9086	0.9631	0.9512	0.9545	0.9905
30	0.8841	0.8875	0.9586	0.9268	0.9302	0.9857
40	0.8734	0.8780	0.9579	0.9024	0.9091	0.9833
50	0.8868	0.8909	0.9632	0.9024	0.9091	0.9833
60	0.8798	0.8843	0.9536	0.8780	0.8889	0.9714
70	0.8915	0.8960	0.9617	0.9268	0.9302	0.9857
80	0.8930	0.8962	0.9622	0.9268	0.9302	0.9833
96	0.8751	0.8804	0.9575	0.9024	0.9091	0.9786

#### 5.2.1 Consistency and reliability analysis of XAI methods

To assess the reliability of the proposed explanation approach, the feature attribution results of three widely used XAI methods, namely DeepSHAP, Integrated Gradients (IG), and Occlusion, were further compared in the EEGPT latent space. As shown in Table 5, the three methods exhibited high consistency, with Spearman correlation coefficients exceeding 0.94 for the importance rankings of all 512 latent dimensions. The Jaccard overlap of the top-20 salient dimensions reached a maximum of 0.905, indicating substantial agreement in the identification of the key discriminative features. These results demonstrate the stability and reproducibility of the core discriminative representations identified by the model. Given its high consistency and its ability to provide more informative feature-attribution values, DeepSHAP was selected for subsequent latent-representation analysis and neurophysiological mapping.

**Table 5:** Comparison of feature-attribution consistency across XAI methods.

Comparison	Spearman ρ	Top-20 overlap	Top-20 Jaccard
DeepSHAP vs Occlusion	0.948	19/20	**0.905**
DeepSHAP vs IG	0.950	17/20	0.739
IG vs Occlusion	0.985	18/20	0.818

### 5.3 Ablation study

#### 5.3.1 Contribution of the handcrafted feature branch

To quantify the contribution of the handcrafted feature extraction branch to overall model performance, two comparative experiments were designed. Specifically, the classification performance of the baseline model that relies solely on the EEG Latent Feature Branch was compared to that of the complete model that integrates the handcrafted feature branch. This comparison aimed to evaluate the practical value of incorporating manually extracted neurophysiological features.

As summarized in Table 6a, incorporating the neurophysiological features extracted by the HFB substantially improved the classification performance with respect to accuracy, F1-score, and sensitivity at both the epoch and subject levels, although this improvement was accompanied by a reduction in specificity. At the epoch level, the EEGPT-Fusion model increased the accuracy from 0.6716 to 0.7532, while the F1-score increased from 0.5787 to 0.7578 and the sensitivity increased from 0.4424 to 0.7569. Meanwhile, the AUC increased modestly from 0.8108 to 0.8273. In contrast, the specificity decreased from 0.9101 to 0.7494, suggesting that the incorporation of handcrafted neurophysiological features enhanced the model’s sensitivity to MDD-related patterns at the expense of reduced specificity.

**Table 6:** Ablation results of core modules.

(a) Handcrafted-feature branch						
Level	Variant	ACC	F1	AUC	SEN	SPE
Epoch-level	EEGPT-only	0.6716	0.5787	0.8108	0.4424	0.9101
	EEGPT-fusion	0.7532	0.7578	0.8273	0.7569	0.7494
Subject-level	EEGPT-only	0.5882	0.3636	0.8472	0.2222	1.0000
	EEGPT-fusion	0.8235	0.8421	0.8472	0.8889	0.7500

(b) Representation-alignment module						
Variant	Overall	Decision	Feature	direction	Mechanism	Consistency
With alignment	100.0	100.0	100.0	100.0	100.0	100.0
Without alignment	85.0	100.0	64.7	94.1	66.7	100.0
Δ	−15.0	0.0	−35.3	−5.9	−33.3	0.0

(c) Quality-control module						
Variant	Overall	Structure	Terminology	Evidence	Logic	No leak
With QC	100.0	100.0	100.0	100.0	100.0	100.0
Without QC	99.4	100.0	100.0	96.1	100.0	100.0
Δ	−0.6	0.0	0.0	−3.9	0.0	0.0

(d) Evidence-guided prompt				
Variant	Quality	Faithfulness	Feature	Mechanism
Evidence prompt	100.0	95.5	79.4	95.9
Normal prompt	86.0	40.0	0.0	0.0
Δ	−14.0	−55.5	−79.4	−95.9

At the subject level, the effect of HFB integration was more pronounced. The EEGPT-Fusion model improved the accuracy from 0.5882 to 0.8235 and the F1-score from 0.3636 to 0.8421, while the sensitivity increased substantially from 0.2222 to 0.8889. In contrast, the specificity decreased from 1.00 to 0.75, whereas the AUC remained unchanged at 0.8472. These findings indicate that the incorporation of handcrafted neurophysiological features markedly enhanced subject-level MDD detection, particularly in terms of sensitivity and overall classification balance, while introducing a trade-off between sensitivity and specificity.

The above results demonstrate that neurophysiological indicators provide complementary physiological information beyond high-dimensional EEG representations, enabling the MDD classification model to achieve more balanced predictions and improved robustness.

#### 5.3.2 Contribution of representation alignment

To investigate the necessity of the Representation Alignment mechanism in the evidence construction stage, an ablation experiment was conducted. Specifically, the Representation Alignment module was removed to construct a control model while keeping all other components and parameters unchanged. The impact of removing this module on the faithfulness of the generated explanations was quantitatively evaluated using standardized automatic assessment metrics.

The changes in the evidence chain before and after removing the representation alignment module are illustrated in Figure 5. As shown in the figure, removing the alignment-based evidence support reduced the consistency between the generated explanations and the underlying discriminative features, while substantially weakening the traceability from EEG latent representations to neurophysiological mechanisms. The quantitative results are presented in Table 6b. After removing the representation alignment module, the overall faithfulness score decreased from 100.0 to 85.0, corresponding to a reduction of 15.0 points. The most pronounced decreases were observed in Feature Alignment and Mechanism Traceability, which declined by 35.3 and 33.3 points, respectively. Evidence Direction Fidelity decreased by 5.9 points, whereas Decision Alignment and Consistency remained unchanged at 100.0.

**Figure 5:**
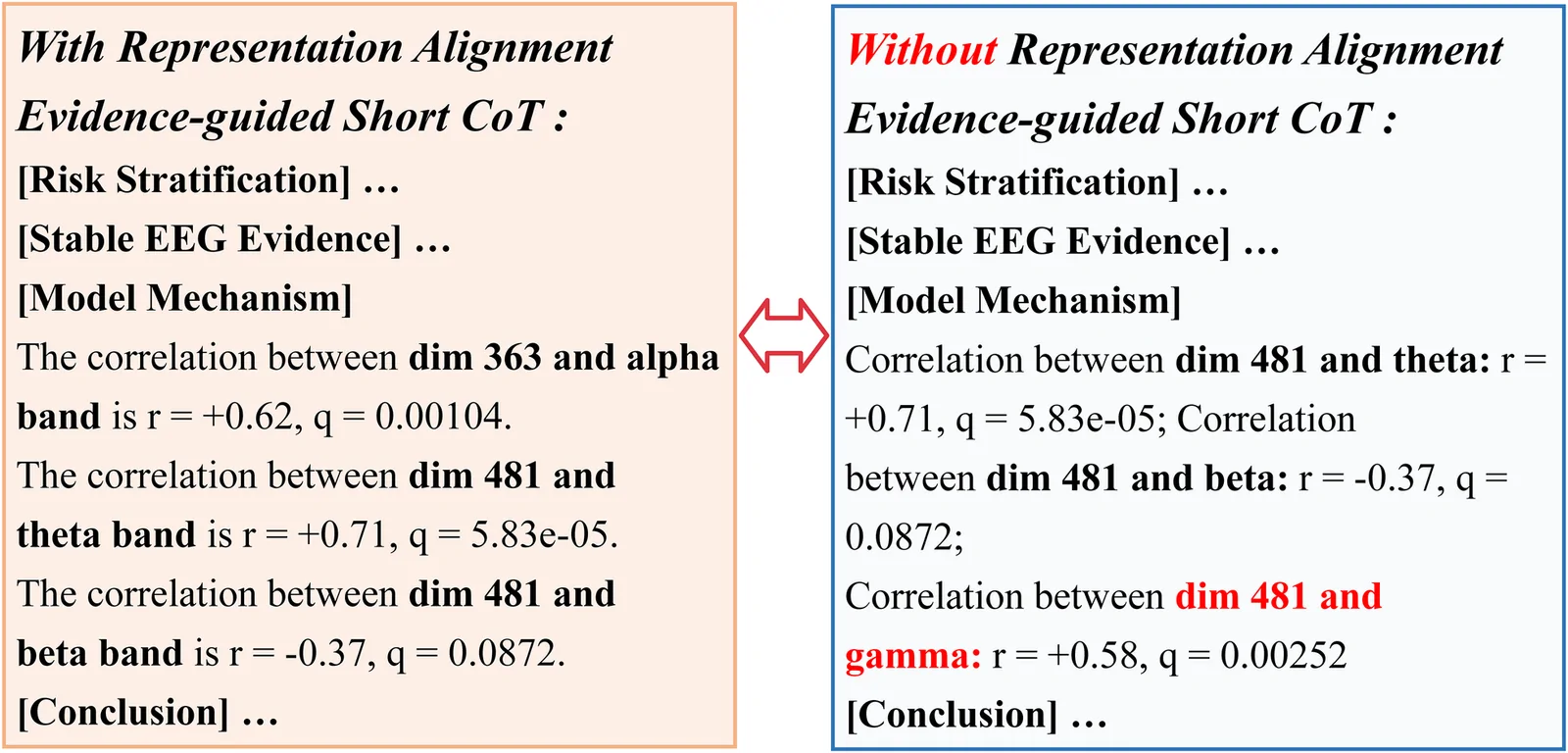
Comparison of the representation alignment module with and without the representation alignment module.

These findings indicate that the representation alignment module primarily establishes explicit associations between high-dimensional EEG features and their corresponding neurophysiological evidence, thereby strengthening feature-level evidence grounding and mechanistic traceability, while exerting a limited influence on the consistency of the final risk decision. In the absence of this alignment mechanism, the evidence-guided explanations remained linguistically fluent and logically coherent but became substantially decoupled from the neurophysiological evidence underlying the model’s decisions, resulting in reduced neurophysiological consistency.

#### 5.3.3 Contribution of the quality control module

To investigate the practical role of the automatic quality control module in the evidence construction process, an ablation experiment was conducted. By comparing the integrity of the generated evidence chains and the quality of the generated explanations before and after removing this module, the optimization effect of the quality filtering mechanism was objectively evaluated.

A representative example of the generated evidence chain before and after removing the QC module is shown in Figure 6. As illustrated in the figure, after removing the QC module, the generated evidence chain continued to reference evidence items labeled as “excluded.” This finding indicates that, in the absence of QC constraints, the explanation generation process may incorporate unreliable or invalid evidence. The quantitative results are presented in Table 6c. Following removal of the QC module, the overall quality-compliance score decreased slightly from 100.0 to 99.4, with the primary change observed in Evidence Coverage, which decreased from 100.0 to 96.1. The remaining metrics, including Structure Completeness, Terminology Consistency, Logical Consistency, and Information Leakage, remained unchanged at 100.0. Taken together, these results demonstrate that the QC module plays an important role in filtering low-quality evidence and ensuring that the generated explanations are grounded exclusively in valid evidence that satisfies the predefined criteria.

**Figure 6:**
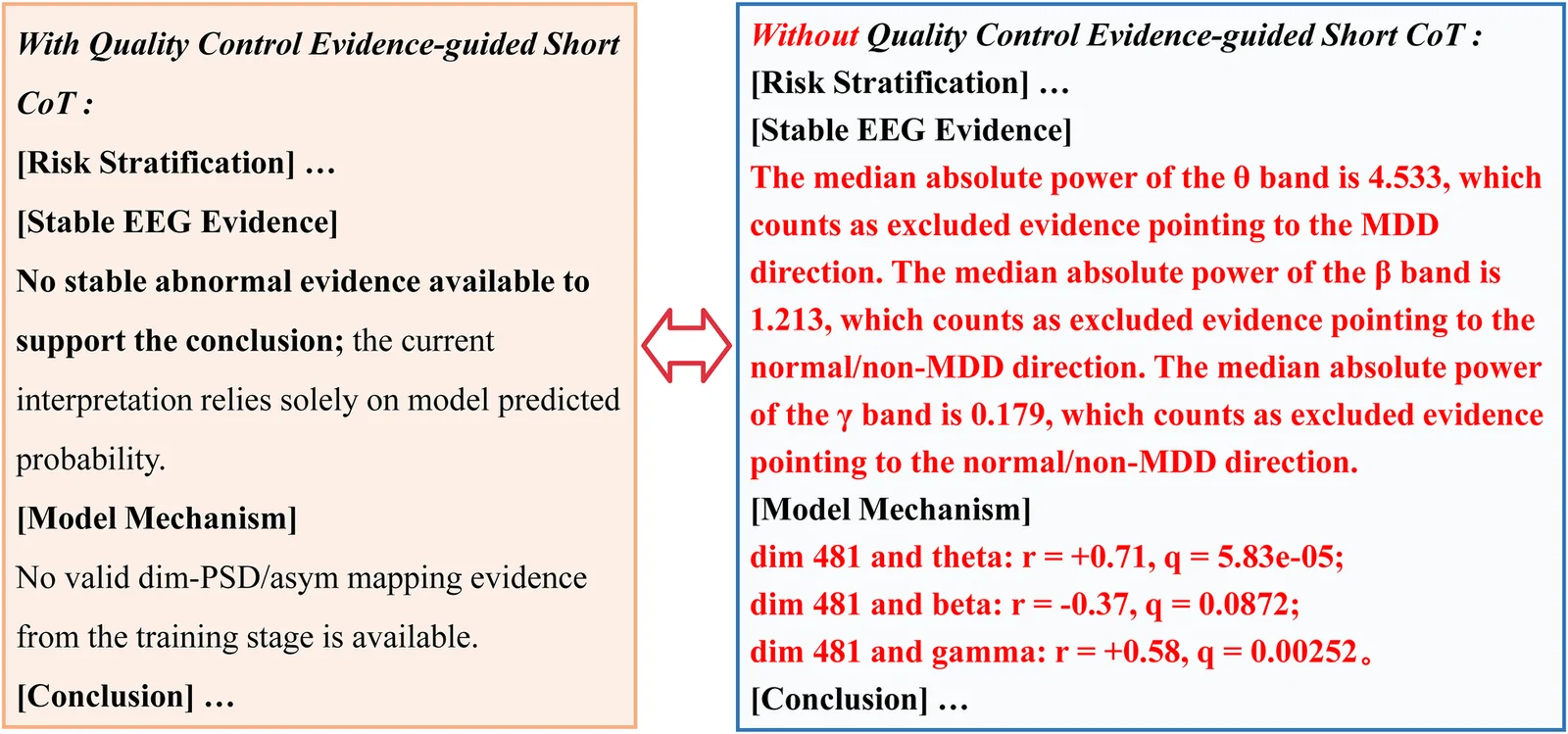
Comparison of the quality control module with and without the quality control module.

#### 5.3.4 Contribution of the evidence-guided prompt

The evidence-guided prompt is designed to constrain the large language model to generate explanations based on structured evidence, thereby improving the faithfulness, consistency, and traceability of the generated explanations. To evaluate its effectiveness, an ablation experiment was conducted by replacing the proposed evidence-guided prompt with a conventional general-purpose prompt while keeping the LLM architecture and inference parameters unchanged. The explanation performance under the two prompting strategies was then compared to demonstrate the effectiveness of the customized evidence-guided prompt.

As shown in Table 6d, replacing the evidence-guided prompt with a normal prompt produced a substantial decline in evidence faithfulness. The overall faithfulness score decreased from 95.5 to 40.0, representing a 55.5-point reduction. Feature Alignment and Mechanism Traceability showed the largest changes, decreasing from 79.4 to 0.0 and from 95.9 to 0.0, respectively. Meanwhile, the quality-compliance score decreased from 100.0 to 86.0 but remained relatively high. This result demonstrates that maintaining a report-like output format does not ensure evidence grounding, whereas explicitly providing biomarkers and latent dimension–biomarker mappings enables substantially stronger feature-level alignment and mechanism traceability.

Representative output examples before and after removing the evidence-guided prompt are illustrated in Figure 7. As shown in Figure 7, compared with the general-purpose prompting strategy, the proposed evidence-guided prompt enables the generated evidence chains to incorporate stable EEG evidence and dim-level biomarker relationships, allowing the final four-part short-text explanations to explicitly associate model decisions with neurophysiologically meaningful supporting evidence. After removing the evidence-guided prompt, the generated outputs were limited to prediction probabilities and risk stratification, without incorporating stable EEG evidence or latent dimension–biomarker associations. Consequently, the generated explanations exhibited insufficient evidence utilization and weaker consistency with the underlying decision process. In contrast, the evidence-guided prompt improved the structural completeness, evidence coverage, and physiological interpretability of the generated explanations, particularly enhancing the alignment between explanations and discriminative features, as well as the traceability from latent EEG representations to corresponding neurophysiological mechanisms. These results demonstrate that the evidence-guided prompt plays a critical role in constraining large language models to generate reliable, evidence-grounded, and physiologically interpretable explanations.

**Figure 7:**
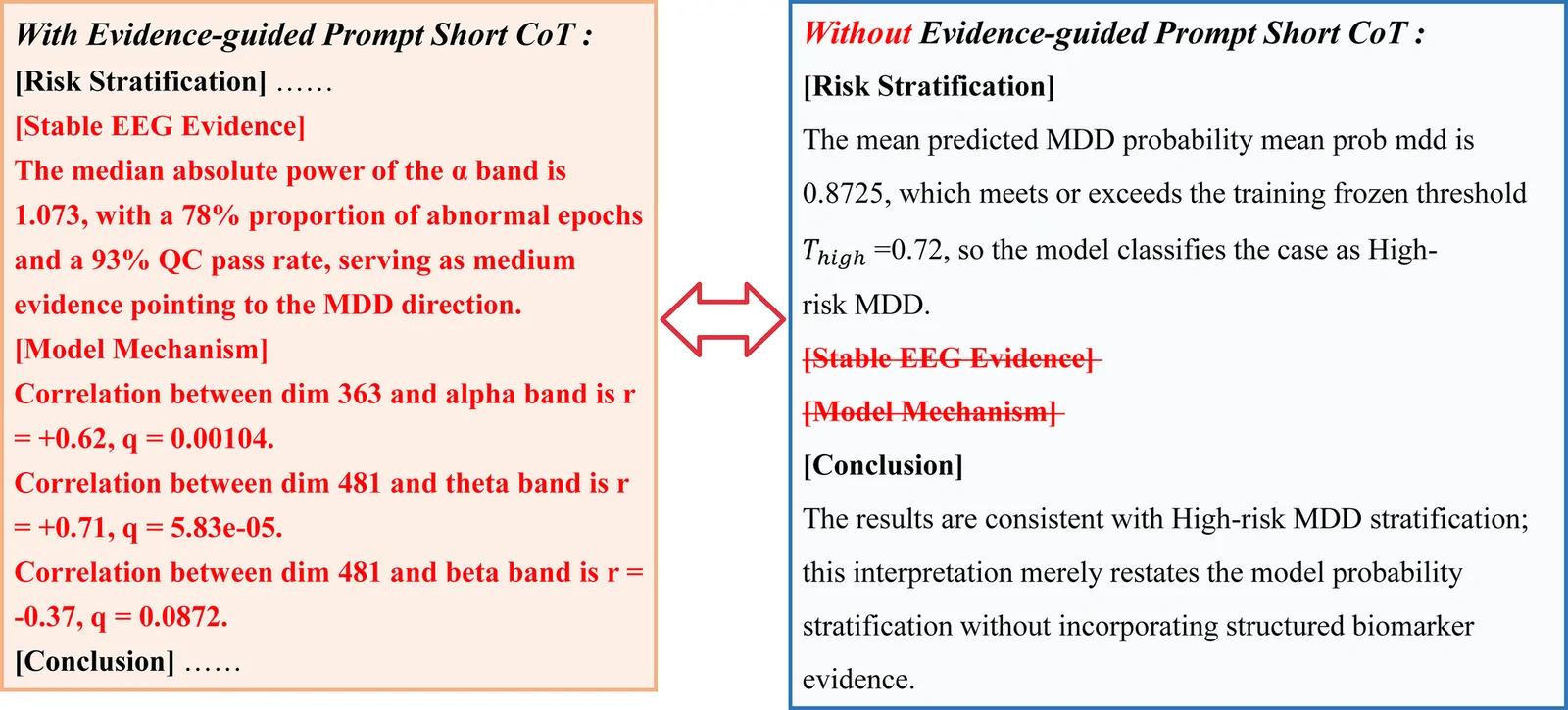
Comparison of generated short-text explanations with and without the evidence-guided prompt.

The results confirm that it is essential to explicitly incorporate structured physiological evidence into the initiation process. Only by providing such evidence guidance can the LLM generate faithful explanations that align with the actual decision-making basis of the model, rather than producing generalized descriptions based solely on common medical knowledge.

Overall, the ablation results demonstrate that the individual components provide complementary contributions to the proposed framework from different perspectives. Their integration enables the proposed framework to jointly support reliable EEG-based depression detection and neurophysiologically grounded interpretation.

## 6 Conclussion

Deep learning techniques based on EEG signals can automatically extract discriminative high-level representations from neural activity patterns, demonstrating significant potential for intelligently assisted depression identification. However, existing studies have focused mainly on improving classification accuracy to enhance detection performance, while largely overlooking the neurophysiological interpretation of high-dimensional representations learned by deep models. Consequently, the intrinsic relationships between latent representations and EEG biomarkers remain not adequately explored, making it difficult to provide neurophysiologically grounded explanations for model decision-making mechanisms. Meanwhile, LLMs have demonstrated remarkable capabilities in natural language understanding and text generation, providing a promising technical pathway for improving the interpretability of intelligent EEG analysis. However, existing approaches that integrate EEG signals with LLMs predominantly generate interpretive explanations directly from raw EEG inputs, without establishing a structured neurophysiological evidence framework grounded in the actual decision-making process of the model. This limitation may lead to a discrepancy between the generated explanations and the underlying reasoning mechanism of the model, resulting in insufficient credibility and limited traceability. Consequently, these approaches remain inadequate to meet the rigorous interpretability and reliability requirements of research-oriented clinical interpretive analysis.

To overcome the limitations of existing approaches, this study proposes an evidence-driven explainable EEG-based depression recognition framework that systematically integrates high-dimensional deep EEG representations, neurophysiological biomarkers, and semantic reasoning capabilities of LLMs within a unified analytical pipeline. The proposed framework establishes a representation alignment mechanism between high-dimensional EEG representations and EEG neurophysiological biomarkers, thus bridging the mapping relationship between latent model representations and explicit physiological indicators. Furthermore, leveraging a structured evidence framework and an evidence-guided prompting strategy, the framework constrains the reasoning generation process of LLMs, allowing strong consistency between depression classification outcomes and their underlying neurophysiological interpretations. This approach effectively alleviates the black-box nature of conventional deep learning models and substantially improves the transparency, reliability, and interpretability of the EEG-based decision-making process.

Experimental results demonstrate that the proposed framework not only achieves superior depression classification performance but also enables precise semantic alignment between high-dimensional EEG representations and neurophysiological biomarkers. The fusion strategy that combines interpretable neurophysiological features extracted from the Handcrafted Feature Branch with deep, high-dimensional representations learned by the model further compensates for the lack of physiological semantics in purely deep feature-based approaches, thus improving both classification accuracy and model robustness. Furthermore, the evidence-driven explanation module generates structured and readable natural language explanations that are strongly aligned with the model-derived evidence, providing a novel paradigm for the recognition of EEG-based depression by jointly optimizing classification performance and neurophysiological interpretability. Despite the promising performance, this study has several limitations that warrant further investigation. First, the subject-level test set was relatively small, consisting of only 17 subjects, which limits the statistical reliability and generalizability of the subject-level performance estimates. Although subject-level data partitioning and subject-clustered bootstrap resampling were adopted to prevent information leakage and quantify statistical uncertainty, the relatively wide 95% confidence intervals indicate that the reported subject-level performance should be interpreted with caution. In particular, the current sample size is insufficient to establish the clinical generalizability of the proposed framework. Future studies will focus on validating the proposed framework using larger, independent, and multicenter EEG datasets to systematically assess its robustness and cross-center generalizability. Second, the framework focuses primarily on spectral neurophysiological biomarkers, and its ability to capture more complex pathological mechanisms underlying brain dysfunction remains limited. Furthermore, incorporating more diverse neurophysiological measurements, such as functional connectivity, brain network topology, EEG microstates, and cross-frequency coupling, together with clinical prior knowledge bases and multimodal patient information, may enable the development of a more comprehensive and fine-grained neurophysiological reasoning framework. Third, the absence of independent clinician or domain-expert evaluation limits the assessment of the clinical relevance, correctness, and practical utility of the generated explanations. Future studies will integrate richer neurophysiological and clinical evidence with independent expert evaluation to further assess the clinical applicability and explanatory validity of the proposed framework.

In general, this study extends the interpretability analysis of deep learning-based EEG models beyond conventional feature attribution approaches toward controllable explanation generation based on neurophysiological evidence. By establishing a complete closed-loop evidence chain from high-dimensional representation extraction and neurophysiological biomarker mapping to interpretable natural language generation, the proposed framework provides a novel and feasible paradigm for provides a potential paradigm for integrating deep learning models and LLMs in interpretable EEG-based decision support.

## Data Availability

The original contributions presented in the study are included in the article/Supplementary Material, further inquiries can be directed to the corresponding author.
